# Restoring Motor Neurons in Spinal Cord Injury With Induced Pluripotent Stem Cells

**DOI:** 10.3389/fncel.2019.00369

**Published:** 2019-08-16

**Authors:** Matthew Trawczynski, Gele Liu, Brian T. David, Richard G. Fessler

**Affiliations:** Department of Neurosurgery, Rush University Medical Center, Chicago, IL, United States

**Keywords:** neural cells, motor neurons, spinal cord injury, induced pluripotent stem cells, transplantation

## Abstract

Spinal cord injury (SCI) is a devastating neurological disorder that damages motor, sensory, and autonomic pathways. Recent advances in stem cell therapy have allowed for the *in vitro* generation of motor neurons (MNs) showing electrophysiological and synaptic activity, expression of canonical MN biomarkers, and the ability to graft into spinal lesions. Clinical translation, especially the transplantation of MN precursors in spinal lesions, has thus far been elusive because of stem cell heterogeneity and protocol variability, as well as a hostile microenvironment such as inflammation and scarring, which yield inconsistent pre-clinical results without a consensus best-practice therapeutic strategy. Induced pluripotent stem cells (iPSCs) in particular have lower ethical and immunogenic concerns than other stem cells, which could make them more clinically applicable. In this review, we focus on the differentiation of iPSCs into neural precursors, MN progenitors, mature MNs, and MN subtype fates. Previous reviews have summarized MN development and differentiation, but an up-to-date summary of technological and experimental advances holding promise for bench-to-bedside translation, especially those targeting individual MN subtypes in SCI, is currently lacking. We discuss biological mechanisms of MN lineage, recent experimental protocols and techniques for MN differentiation from iPSCs, and transplantation of neural precursors and MN lineage cells in spinal cord lesions to restore motor function. We emphasize efficient, clinically safe, and personalized strategies for the application of MN and their subtypes as therapy in spinal lesions.

## Introduction

Spinal cord injury (SCI) results in a debilitating loss of motor, sensory, and autonomic function. SCI comprises two phases of injury: the initial mechanical damage to sensorimotor tracts and vasculature and the secondary inflammatory-ischemic cascade, leading to infarction and scarring (Rowland et al., 2008). Many treatments have been proposed to address both phases, including surgical decompression and fusion, pharmacotherapy and hypothermia (Kwon et al., 2011; Fehlings et al., 2014; Gazdic et al., 2018). Over the past decade, stem cell therapies have also received significant attention because of their potential to restore and/or salvage damaged neurons (e.g., MNs) and glia (e.g., astrocytes, oligodendrocytes, Schwann cells, and microglia). In particular, stem cell-derived MNs and MN-progenitors are promising potential regenerative transplant therapies because even small changes in engraftment growth with endogenous, injured neurons can have significant effects on motor recovery, and quality of life (Bonner and Steward, 2015). Thus, repairing MN pathways is a critical step to restoring quality of life for individuals with SCI.

Many different types of stem cells have been transplanted in SCI, varying by source (e.g., embryonic and adult) and stage of differentiation (e.g., pluripotent and multipotent). ESC transplants have been the most studied because of their plasticity, self-renewal, *in vivo* survival and integration, neurotrophic capabilities, and well-established capability to differentiate into MNs and glia (McDonald et al., 1999; Lee et al., 2007; Peljto et al., 2010; Curtis et al., 2018; Rosenzweig et al., 2018). However, ESCs raise ethical and immunogenic concerns. Mesenchymal stem cells (MSCs) have also been shown to differentiate into MNs, using cells harvested from bone marrow (Ma et al., 2011; Faghihi et al., 2015), umbilical cord blood (Yousefi et al., 2017), and adipose tissue (Darvishi et al., 2017). However, MSC transplants have been found to yield inconsistent results (Gowing and Svendsen, 2011; Oliveri et al., 2014; Qu and Zhang, 2017) and mature MSC-MN subtypes are not well characterized. The selection of MSC source (bone marrow, umbilical cord blood, and adipose) can significantly affect differentiation efficiency and cell fate because of ingrained epigenetic memory signatures (Xu et al., 2017). Finally, iPSCs, like ESCs, are characterized by a high degree of plasticity and show promising capacities for mature MN differentiation and transplantation in SCI (Nori et al., 2011; Lu et al., 2014b). Because iPSCs have been used to derive many different MN subtypes (with mitigated ethical and immunogenic concerns) and because ESCs and iPSCs have equivalent differentiation potential into MNs (Marei et al., 2017), iPSC-derived MNs may currently be more promising for clinical application (Nagoshi and Okano, 2018).

Challenges remain, however, in translating these findings to the clinic. These include concerns over methodological differences, variable stages of maturity, differentiation efficiency, scalability, purity, and teratoma formation (Casarosa et al., 2014; Nori et al., 2015). Some of these are already being addressed (Nagoshi and Okano, 2018) and recent advances in single-cell transcriptomics have begun to more precisely define the stages of MN lineage (pluripotent, precursor, MN-committed progenitor, and post-mitotic MN) (Rizvi et al., 2017). Each of these stages is identified by multiple biomarkers as there is no single marker specific to each stage. Consequently, variability exists in the maturity of cells being transplanted. These challenges are further compounded by SCI inter-individual heterogeneity, resulting in different MN subtypes and spinal regions injured with each lesion. Because of this heterogeneity, some individuals may need gray matter grafts (e.g., lower MNs at ventral horn) while others may receive white matter grafts (e.g., upper MNs at descending tracts, lower MNs at anterolateral sulcus) – adding further variables that need to be taken into account. For these reasons, it is crucial to standardize novel experimental techniques for deriving neural precursors and MN subtypes from iPSCs for the repair of MN pathways in SCI.

In this review, we aim to address this need by summarizing the biological mechanisms, current techniques and derivation methods for generating MNs and MN subtypes, and SCI transplantation applications of iPSC-MN lineage cells. In an effort to maximize protocol standardization and clarity, we review each of these in relation to three levels of MN differentiation: neural precursors, cells committed to a MN-lineage, and mature MN subtypes. We emphasize recent techniques which are efficient, scalable, MN subtype-specific, and clinically-relevant. Of particular significance, we discuss the challenges of targeting specific MN subtypes in SCI, as well as strategies for directly or indirectly replacing damaged MN subpopulations through personalized SCI transplantations.

## Mechanisms

### Induced Pluripotent Stem Cells

Pluripotent stem cells are defined by a capacity for unlimited cell renewal and differentiation – both *in vivo* and *in vitro*. PSCs are able to differentiate into endoderm, mesoderm, and ectoderm fates. Ectoderm consists of the neural tube and neural crest and will differentiate into a neural fate. Mesoderm differentiates into muscle and bone, while endoderm becomes luminal epithelia. Both ESCs and iPSCs have been found to have equivalent differentiation potential into MNs, likely driven by highly similar mRNA profiles (Marei et al., 2017). ESCs in particular are derived from the developing embryo at the blastocyst stage and, although generally identified in the inner cell mass, they can be harvested from any developmental stage before implantation (Boroviak et al., 2014). iPSCs are adult cells which have been reprogrammed by the forced expression of pluripotent transcription factors; these classically include SOX2, OCT4, KLF4, and c-Myc among others. The process of epiblast specification, in particular, has been implicated in the induction of pluripotent characteristics, namely self-renewal. Some studies have also identified pluripotent-like stem cells residing in adult bone marrow (Ratajczak et al., 2008), but these findings remains controversial. Both ESCs and iPSCs are characterized by their expression of markers of pluripotency, most commonly SOX2, OCT4, undifferentiated embryonic cell transcription factor 1 (UTF1), reduced expression protein 1 (REX1), alkaline phosphatase, and human telomerase (Kumar et al., 2015). The specific molecular mechanisms surrounding pluripotency have been reviewed previously (Lin, 2011; Chhabra, 2017).

#### Neural Precursors

**FIGURE 1:**
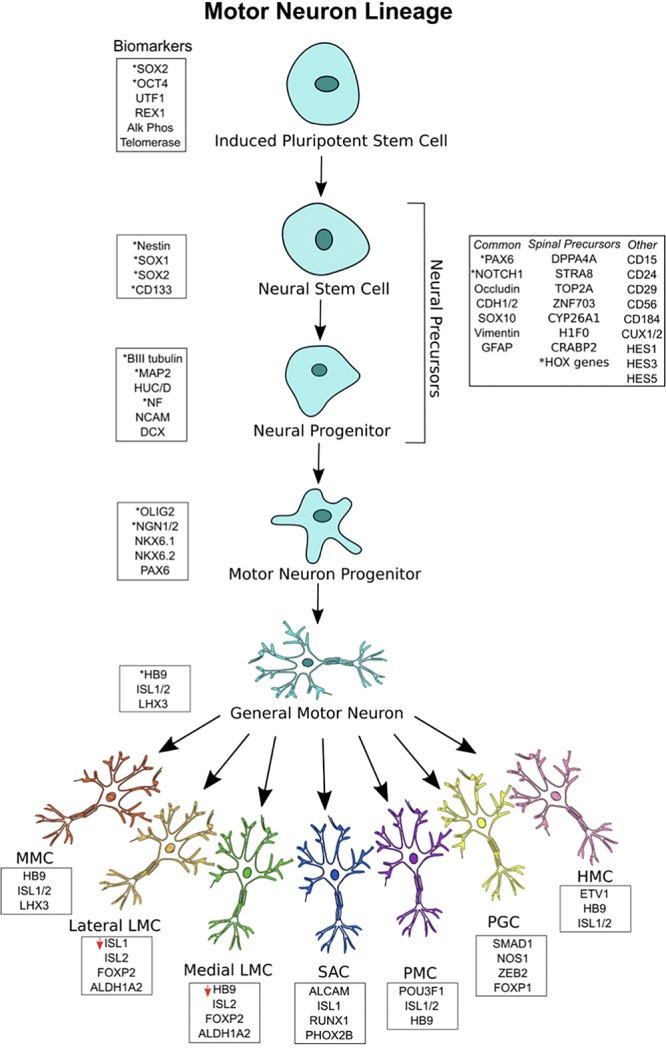
Motor neuron (MN) lineage with gene expression at each stage. iPSCs express canonical markers of pluripotency, and neuroectodermal patterning generates a heterogeneous pool of neural stem cell and neural progenitors, termed neural precursors. Significant overlap exists between biomarkers for the different stages. General MNs are defined as mature MNs expressing HB9, ISL1/2, and LHX3, showing electrophysiological and synaptic activity. General MNs undergo further patterning to form MN subtypes, each with its own rostral-caudal position and gene signatures. Lateral LMC MNs have decreased ISL1 expression, while medial LMC MNs have deceased HB9 expression. All MN subtypes overlap in their expression of HB9, ISL1/2, and LHX3. ^∗^Asterisks represent the most commonly used biomarkers.

During embryogenesis, PSCs are guided by signaling molecules and transcription factors through intermediate stages to their end cell fate, after which they mature and perform a variety of functions based on their specification and anatomical location. The mechanisms regulating neural induction, caudalization, ventralization, differentiation, specification, and maturation into functional spinal MNs have previously undergone in-depth review by Jessell (2000), more recently, by Stifani (2014) and Davis-Dusenbery et al. (2014). The timeline of MN lineage is depicted in Figure 1. In brief, early experiments established TGFβ and FGF as direct inducers of mesodermal lineage through mothers against decapentaplegic-family proteins (SMAD), leading to muscle and connective tissue cell fates. Neural fate, however, is derived from the ectodermal germ layer. Inhibiting TGFβ and FGF causes the ectodermal neural plate to fold inward and roll into the neural tube, which is the embryonic form of the spinal cord and brain (Ozair et al., 2013). Endodermal fate may also be induced from iPSCs, leading to lumen epithelia, gallbladder, and pancreatic fates. Another way to induce a neural fate is through direct neural-inducing enzymes (i.e., noggin, chordin, and follistatin) which inhibit TGFβ-family ligands such as BMP, growth differentiation factor (GDF), activin, and nodal growth differentiation factor (NODAL) among others. PSCs treated with these factors undergo neuralization. Multiple small-molecule neural-inducers and inhibitors of TGFβ were also identified, including SB431542 and DMH1. Taken together, these factors promote neural induction; however, a forebrain identity is assumed without further signaling molecules. Thus, to form a spinal cell identity, a rostral-caudal identity is specified next. The factors RA, Wnt, and FGF induce a caudal, posterior identity, resulting in the generation of neural precursors with a distinctly spinal character, which will ultimately form lower MNs. RA contributes to rostral, cervical fates while GDF11 and FGF contribute to thoracic and lumbar fates of neural precursors (Diez del Corral and Storey, 2004; Liu, 2006; Patani, 2016).

Neural precursors refer to a heterogeneous pool of neural stem cells, neural progenitor cells, neuroepithelial cells, radial glial cells, and other neural intermediates – each existing in a different stage of differentiation on its way to a MN fate. Because there is no singular marker specifying neural precursors or stem cells, they are identified by the expression of a variety of surface markers, receptors, and transcription factors (Jandial et al., 2008). Neural stem cells are well described to express NES, SOX1, SOX2, and CD133 (Gibco, 2012). Neural progenitors, being more limited in their capacity to differentiate and proliferate than neural stem cells, commonly express microtubule associated protein 2 (MAP2), Hu antigen C/D (HUC/D), nuclear factor (NF), neural cell adhesion molecule (NCAM), βIII-tubulin, and doublecortin (DCX). Both neural stem cells and neural progenitors, collectively termed neural precursors, show significant overlap in these markers and may also express the following: paired box 6 (PAX6), cut-like homeobox 1/2 (CUX1/2), Notch 1, hes family bHLH transcription factor 1/3/5 (HES1/3/5), occludin (OCLN), cadherin 1/2 (CDH1/2), SOX10, vimentin (VIM), glial fibrillary acidic protein (GFAP), CD15, CD24, CD29, CD56, and CD184 (Bylund et al., 2003; Jandial et al., 2008; Kageyama et al., 2008; Yuan et al., 2011; Noisa et al., 2014; Zhang and Jiao, 2015). Other markers are expressed by those neural progenitors with a specific spinal identity, including those that have been caudally induced by RA. Single-cell RNA sequencing has revealed some of these markers to include developmental pluripotency associated 5 (DPPA5), stimulated by STRA8, DNA topoisomerase II alpha (TOP2A), zinc finger protein 703 (ZNF703), cytochrome p450 family 26 subfamily A member 1 (CYP26A1), h1 histone family member 0 (H1F0), cellular retinoic acid binding protein 2 (CRABP2), and various HOX genes (Rizvi et al., 2017). Many of these are hallmarks of RA-treatment and thus provide a confirmation of spinal neural progenitor identity.

#### Motor Neuron Progenitors

After neuralization and caudalization, the process of ventralization takes place to generate ventrally-positioned cells of the spinal cord (e.g., lower MNs and interneurons). Graded levels of Shh are responsible for regulating several transcription factors, namely bHLH and HD factors, to induce a more ventral cell identity (Jessell, 2000; Davis-Dusenbery et al., 2014; Stifani, 2014). Shh concentration increases along an increased ventral axis, while Wnt/BMP concentrations decrease along this axis. HD proteins, which are divided into Class I or Class II, interpret signals from Shh to generate sharply-defined ventral progenitor domains. Class I proteins include PAX6, iroquois homeobox 3 (IRX3), and developing brain homeobox 1/2 (DBX1/2). Class II proteins include NKX6.1, OLIG2, and NKX2.2. Cross-repressive activity between Class I and Class II proteins in response to variable Shh signaling delineates specific neural progenitor domains, each with a unique expression pattern of HD proteins. Five unique domains are specified by these signaling mechanisms – p0, p1, p2, p3, and pMN. MNs and oligodendrocytes arise from the pMN domain, while ventral interneuron classes arise from p0–p3. Thus, graded Shh signaling results in the generation of MN progenitors.

The most important markers of MN progenitors specific only to the pMN domain are OLIG2 and NGN1/2 (Jessell, 2000; Davis-Dusenbery et al., 2014; Stifani, 2014; Rizvi et al., 2017). The markers NKX6.1, NKX6.2, and PAX6 are also expressed by these cells, but these overlap between domains. The relationship between OLIG2 and NGN2 is worth noting. With early OLIG2 expression, MN progenitors are still able to differentiate into MNs or oligodendrocytes. NGN2 works to repress OLIG2 and induces a committed post-mitotic MN lineage (Lee et al., 2005). Thus, both are expressed inversely in the MN progenitor domain and, as a result, MNs and oligodendrocytes are both generated from the same domain (Stifani, 2014). As this takes place, a gradient within the pMN domain specifies the most ventrally located progenitors to become MNs, while Shh-mediated pathways recruit neuroepithelial progenitors to the dorsal pMN domain for oligodendrocyte fates (Ravanelli and Appel, 2015). Thus, MN progenitors committed to MN differentiation are located most ventrally in the pMN domain and express OLIG2 and NGN1/2.

#### Post-mitotic Motor Neurons

Following MN progenitor formation, NGN2 is highly expressed, and post-mitotic lower MNs are generated. NGN2 induces the expression of HB9, which works to consolidate MN identity (Arber et al., 1999; Lee et al., 2009; Stifani, 2014). In addition, Shh signaling promotes ISL1 and LHX3 cooperation, which leads to the transcription of genes responsible for acetylcholine-transmitting MNs. In order to promote the migration of post-mitotic MNs, FOXP2/4 causes ventricular zone detachment (Rousso et al., 2012; Stifani, 2014). As a result, the general character of post-mitotic MNs is formed.

After MNs exit the cell-cycle, additional specification occurs. HOX-family genes are differentially expressed along the rostral-caudal axis to form distinct lower MN columns in the cervical, thoracic, lumbar, and sacral spinal cord. The order of HOX chromosomal loci determines HOX gene rostral-caudal expression. Thus, HOX4-8 are expressed in the brachial MN column. Similarly, HOX8/9 are expressed in the thoracic region and HOX10/11 genes are highly expressed in lumbar regions (Dasen and Jessell, 2009; Philippidou and Dasen, 2013; Davis-Dusenbery et al., 2014; Stifani, 2014). This patterning contributes to specifying six lower MN columns along the rostral-caudal axis: the median motor column (MMC), SAC, PMC, LMC, HMC, and the PGC. Each motor column innervates a specific muscle group. MMC neurons innervate axial musculature. SAC neurons innervate neck musculature and PMC neurons innervate the diaphragm. LMC neurons innervate appendage musculature and can be further split into lateral LMC, which innervate dorsal musculature of appendages, and medial LMC, which innervate ventral musculature of appendages. HMC neurons innervate thoracic intercostal/abdominal musculature and PGC neurons connect to sympathetic ganglia. Thus, the many muscle groups in the body are innervated by diverse MN subtypes.

Post-mitotic MNs have distinct gene expression signatures. General MNs are most often defined by HB9 expression, but they also express ISL1, ISL2, and LHX3 (Arber et al., 1999; Sances et al., 2016). MMC MNs express HB9, ISL1/2, and LHX3; SAC MNs express activated leukocyte cell adhesion molecule (ALCAM), ISL1, runt related transcription factor 1 (RUNX1), and paired like homeobox 2b (PHOX2B). PMC MNs express POU3F1, ISL1/2, and HB9; PGC MNs express SMAD family member 1 (SMAD1), nitric oxide synthase 1 (NOS1), zinc finger E-box binding homeobox 2 (ZEB2), and FOXP1. HMC MNs express HB9, ISL1/2, and ETS variant 1 (ETV1); LMC MNs express ISL2, FOXP1, and aldehyde dehydrogenase 1 family member A2 (ALDH1A2) (Tsuchida et al., 1994; Sockanathan and Jessell, 1998; Dillon et al., 2005; Dasen et al., 2008; Rousso et al., 2008; Stifani, 2014). Lateral LMC MNs have been found to underexpress ISL1, while medial LMC MNs underexpress HB9 (Kania and Jessell, 2003; Sockanathan et al., 2003; Rousso et al., 2008). As a result, each lower MN subtype can be identified by unique gene expression signatures.

#### Upper Motor Neurons

The RA/Wnt/FGF pathways reviewed thus far form lower MNs, which are cholinergic, releasing acetylcholine into the synaptic cleft. Upper MNs and upper MN tracts, which include corticospinal, reticulospinal, tectospinal, rubrospinal, and vestibulospinal tracts among others, are glutamatergic, and release glutamate onto lower MNs or interneurons (reticulospinal is both glutamatergic and GABAergic/glycinergic). As we reviewed, the generation of lower MNs occurs in three stages of differentiation: neural precursor, MN-progenitor, and post-mitotic MN. Less is known about the more complex embryogenesis of glutamatergic upper MN tracts and the complex, and often uncharacterized, mechanisms guiding their development and subtyping. Since multiple facets of the differentiation of iPSCs to upper MNs have not been characterized, and since upper MN precursors have not yet been transplanted in models of SCI, we do not focus on upper MNs in this article. The current standard for *in vitro* differentiation of upper MNs yields generic, immature, progenitor-like cortical neurons. These limitations have recently undergone review by Sances et al. (2016). In brief, after neuralization, precursors retain a forebrain identity. In lower MN development, RA, Wnt, and FGF induce a caudal, spinal identity. However, in upper MN development, a rostral identity is established by antagonism of these factors (Watanabe et al., 2005). Rostral and telencephalic identity is specified by OTX2 and FOXG1 expression (Tao and Lai, 1992; Acampora et al., 1999). Further subtype specification and maturation is under transcriptional regulation by fasciculation and elongation protein zeta 2 (FEZ2F), BAF complex component (BCL11B), orthodenticle homeobox 1 (OTX1), sex determining regions Y box 5 (SOX5), POU3F1, and other transcription factors that regulate axonal outgrowth and pruning. This leads to the specification of descending pyramidal MN tracts (Arlotta et al., 2005; Molyneaux et al., 2005; Sances et al., 2016). These genes are signatures of upper MN identity, specifically of a corticospinal fate (Arlotta et al., 2005; Molyneaux et al., 2005; Hansen et al., 2011; Sances et al., 2016). Thus, upper MNs are also identified by these gene signatures.

#### Mechanisms of Motor Neuron Injury in Spinal Cord Lesions

It is well known that certain MN pools have increased susceptibility to MN diseases. As a result, iPSC-derived MN subtypes may be a promising personalized therapy to target certain susceptible MNs. This is because iPSCs have lower ethical and immunogenic concerns than ESCs. In addition, like ESCs, iPSCs are able to differentiate into a wide variety of lower MN subtypes. For example, some MNs have increased susceptibility to ALS – spinal MNs in particular exhibit graded vulnerability to ALS, with fast-twitch MNs affected first (Nijssen et al., 2017). Susceptible MNs in ALS also show an early increased soma size, even after controlling for spinal region, gender, and neuronal type (Dukkipati et al., 2018). Further, corticospinal neurons have increased vulnerability to the pathological mechanisms surrounding endoplasmic reticulum stress (Jara et al., 2015). As a result, targeted replacement of vulnerable MN populations with iPSC-MNs may be a future therapeutic strategy for MN diseases.

In contrast to MN diseases like ALS, significantly less attention is given to differential MN subtype vulnerability in SCI. This is especially relevant considering that a hallmark of SCI is inter-individual heterogeneity. The fine-tuned packaging and organization of neuronal tracts, lower MNs, interneurons, decussations, nerve roots, and microvasculature in the spinal cord allows for inter-subject variability regarding structural damage, dislocation, compression, transection, or concussion. Today, SCI is most often incomplete, meaning that some level of function below the primary injury is retained (Sekhon and Fehlings, 2001). Complete transection is a rare occurrence, and incomplete SCI generally spares some white matter surrounding the core of the lesion (Kramer et al., 2013). These observations suggest that while some neuronal or vascular structures are damaged, others could be relatively preserved.

While the degree of SCI severity plays the primary role in the level of damage that MNs undergo, studies have largely supported the theory that intrinsic differential MN susceptibility may also contribute to SCI severity and recovery of function. For example, the level at which SCI occurs can influence which MN pools are affected. In pediatric populations, lower MNs have been found to have increased vulnerability in caudal lesions (Johnston et al., 2005). Further, lower MN damage has been found to be significantly more common in lesions at spinal cord (neurologic) T10 – T12 levels, while upper MNs are susceptible to damage in spinal cord T7 – T9 lesions (Doherty et al., 2002). Lesions at spinal cord L1 – L3 exclusively damage lower MNs because upper MN columns terminate prior to the cauda equina. Because unique MN subtypes and muscle groups are located at each spinal level, each SCI damages different MN pools (Wilcox et al., 2017). Upper MN tracts have also been found to have differential susceptibilities. Propriospinal and corticospinal tracts, which are well established to reorganize into lesion-circumventing relays (Courtine et al., 2008; Rosenzweig et al., 2010; Filli and Schwab, 2015), have been found to have increased susceptibility in certain models of SCI (Blisard et al., 1995; Siebert et al., 2010; Conta Steencken et al., 2011; Hassannejad et al., 2018). Not only is differential MN vulnerability seen in the primary mechanical phase of SCI, but recent advances have demonstrated that SCI at differing spinal levels is characterized by a level-specific immune response (Hong et al., 2018). This can be measured by plasma cytokine levels, with cervical SCI characterized by decreased systemic immune marker proliferation. Thus, both pathological phases of SCI show unique, level-specific injury hallmarks, which could play a driving role in MN subtype vulnerability.

These observations suggest that personalized iPSC-MN transplants, designed to exploit inter-subject heterogeneity by targeting certain vulnerable or selectively injured MN populations, could be effective therapies in SCI. Recent studies have demonstrated how strategically targeting propriospinal neurons, for example, can lead to robust axonal outgrowth that passes through scarring to establish new connections across lesions (Anderson et al., 2018). Stem cell therapies to replace certain MN subtypes with experimentally derived precursors or neurotrophic signaling could more effectively address the complexities and heterogeneity of SCI (Dell’Anno and Strittmatter, 2017; Iyer et al., 2017). Thus, efficient methods of differentiating MN-precursors or subtypes from iPSCs, reviewed next, could be valuable tools to understand and treat SCI.

## Methods

### Generation of Induced Pluripotent Stem Cells

Thus far MNs have been derived *in vitro* from a variety of PSCs and cell resources. These include PSCs (ESCs and iPSCs) as well as MSCs. ESC-MNs and iPSC-MNs and their MN subtypes are increasingly well characterized and confirmed through downstream electrophysiology and grafting. MSC-MNs, however, have only been poorly characterized, and concerns exist over whether MSCs can be differentiated into mature MNs. Some studies seem to have succeeded in generating MNs from MSCs, but these are not always confirmed with electrophysiology, purity analysis, synapse-formation, or spinal grafting (Faghihi et al., 2015; Darvishi et al., 2017; Yousefi et al., 2017). For these reasons, ESC and iPSC MNs may hold greater clinical potential than MSC MN-like cells. However, since ESCs presenting significant ethical and immunogenic concerns, iPSCs may hold greater clinical potential at this time. Thus, iPSC-MNs, which have been differentiated and confirmed by a variety of protocols and research groups – including advances regarding MN subtyping – could be a promising clinical-grade cell replacement therapy for MN restoration in SCI.

Induced pluripotent stem cells have been generated for MN differentiation through a wide variety of methods, with more recent protocols avoiding animal products and viral genomic integration. iPSCs may be derived from fibroblasts, umbilical cord blood cells, urine, and keratinocytes among other cell types (Raab et al., 2014). These may be reprogrammed using transcription factors delivered by integrating viruses (retrovirus and lentivirus) or non-integrating viruses (adenovirus and Sendai virus). Other methods of clinically safe iPSC derivation methods include various expression plasmids, self-excising vectors like piggyBac transposon or Cre-loxP, synthetic mRNAs, and methods of chemical induction (Malik and Rao, 2013). These methods aim to induce expression of pluripotency-associated transcription factors, namely combinations of SOX2, OCT4, KLF4, c-Myc, Nanog homeobox (NANOG), and lin-28 homolog A (LIN28) (Kumar et al., 2015). They have previously undergone in-depth analysis (Malik and Rao, 2013; Zhou and Zeng, 2013; Raab et al., 2014).

To date most iPSC-MN protocols have generated iPSCs using integrating viral delivery of transcription factors to human fibroblasts. Of the ten iPSC-MN protocols we review here, two employed non-integrating Sendai virus, with the rest using retroviruses, or lentiviruses (Table 1). The selection of iPSC induction method is critical to downstream clinical application, and advances in clinically-safe iPSC induction (synthetic mRNAs, self-excising vectors, etc.) have not yet been used to their full potential to generate MNs. This being said, studies are currently underway to develop clinically-safe iPSC lines with the goal of enabling future clinical trials to transplant neural precursors into SCI (Tsuji et al., 2010; Nagoshi and Okano, 2018).

**TABLE 1:** Summary of MN differentiation protocols from induced pluripotent stem cells.

**Year**	**Protocol**	**iPSC**	**NPC**	**MN progenitor**	**Mature MN**	**MN subtype**	**Electrophysiology**	***In vitro* NMJ**	***In vivo* integration**	**Animal product free**	**Duration (iPSC to MN)**	**Efficiency (%)**
2009	Chambers et al., 2009	Lentivirus; fibroblasts	Pax6, Nestin	–	Isl1, HB9	–	–	–	–	No	∼19 days	–
2009	Karumbayaram et al., 2009	pMX retrovirus; fibroblasts	Brn2, Sox3, Pax6, Nestin	Nkx6.1, Olig2	βIII-tubulin, ChAT, ISL1, HB9	Cervical	Induced patch clamp	–	–	No	∼40 days	∼30
2011	Hester et al., 2011	Retrovirus; fibroblasts	PSA-NCAM, Otx2	–	ChAT, HB9	Cervical, thoracic	Induced/Spontaneous patch clamp	Yes	–	No	11–30 days	49–72
2013	Amoroso et al., 2013	–	βIII-tubulin	–	HB9, Isl1, Isl2, NEFL, RET, CHAT, CHT1, VACHT, CHRNA3, CHRNA4, CHRNB2, FOXP1, LHX3, RALDH2	Brachial, LMC (m/l)	Induced patch clamp	–	Chick embryos	No	∼21 days	50
2014	Qu et al., 2014	Lentivirus; fibroblasts	Pax6, Sox1, Zic1	–	βIII-tubulin, HB9	–	Induced patch clamp	Yes	–	No	∼20 days	∼30–52
2015	Maury et al., 2015	Retrovirus; fibroblasts	Pax6, Nestin	NKX6.1, Olig2	HB9, ISL1, ChAT, Foxp1	LMC	Induced patch clamp	Yes	–	No	∼14 days	∼77
2015	Du et al., 2015	Sendai; fibroblasts	Sox1, Hoxa3, Otx2	Olig2	Mnx1, Isl1, ChAT	–	Induced patch clamp	Yes	Chick embryos	No	∼28 days	∼90
2015	Lippmann et al., 2015	Lentivirus; fibroblasts	Pax6, Sox2, HOX1-9, CDX1, CTNNB1,	–	βIII-tubulin, Isl1, SMI-32, synapsin, HB9	Potentially all subtypes: cervical, thoracic, lumbar	Induced/Spontaneous patch clamp	–	–	No	∼21 days	–
2017	Goparaju et al., 2017	Sendai virus/retrovirus; fibroblasts	Tubb3, Tkda3-4, Map2, NeuN	–	Isl1, HB9, ChAT	–	Induced patch clamp	–	–	Yes	∼7–14 days	94–100
2017	Lukovic et al., 2017	pMX retrovirus; fibroblasts	Pax6, Sox1, Zic1, Zo1, Musashi, Bf1, OTX2, A2B5, Nestin, Sox2, βIII-tubulin, Dach1	–	Isl1, Isl2	Cervical	Induced/Spontaneous patch clamp	–	Mouse striatum	Yes	∼49 days	–

*Note the increased efficiency and yield with more recent protocols. Lukovic et al. (2017) developed an EB-free, animal-free, feeder-free, but relatively lengthy (49 days) method to generate iPSC-MNs. The combination of this method with non-integrating iPSCs would improve clinical applicability and result in the best protocol for clinical applications developed thus far. Other methods minimized protocol duration [Goparaju et al. (2017) in 7–14 days] or maximized scalability, generating iPSC-MNs in 384-well plates Maury et al. (2015). Research groups interested in differentiating particular MN subtypes may consider the method developed by Lippmann et al. (2015) which allows for the generation of a wide variety of MN subtypes along the rostral-caudal axis by varying morphogen treatment timing. These methods represent the evolution of iPSC-MN differentiation strategies, with more recent protocols increasing in efficiency, scalability, and clinical applicability.*

It is also well know that iPSCs and iPSC-derived cells have limitations, especially in the context of culture heterogeneity, dosage variability, debate over best route of administration, and survivability in the hostile inflammatory SCI microenvironment. Recent advances have begun to unravel the physiological and experimental reasons for this variation. Advances in single-cell RNA sequencing have demonstrated that certain loci of interest, termed eQTLs, as well as Polycomb genes bear some responsibility for inter-donor batch genetic and epigenetic variability (Carcamo-Orive et al., 2017). Other limitations of iPSCs are currently being investigated (Nagoshi and Okano, 2018), and some studies have shown intralesional transplants to be most beneficial when compared with other sites of implantation, especially in ESCs (Takahashi et al., 2011). It is also well known that the SCI microenvironment is hostile to cell growth, and transplanted iPSC-derived cells may have difficulty integrating with existing neuronal networks. These challenges are being addressed by promoting immune suppression, either pharmacologically, by transplanting cells with immunomodulatory properties, or by varying the timing of transplantation post-SCI (Rossi et al., 2010; Nori et al., 2017). Regardless, future studies will need to determine why these limitations occur and how they may be overcome in therapeutic SCI transplants.

### Differentiation of Neural Precursors

Once iPSCs have been generated, the next step in MN differentiation is the induction of a neural fate. To date, a variety of methods have been used to generate neural precursors for MN differentiation. Neural precursor formation is a critical first step to generating MNs *in vitro*, and methods include embryoid body (EB) formation in feeder culture, monolayer differentiation, and viral or synthetic mRNA delivery of transcription factors – each of which can be combined with small-molecule neural induction. These methods vary in efficiency, clinical applicability, and duration. These methods have undergone significant revision over the past decade with the goal of clinical translation because they need to be scalable, efficient, safe, non-tumorigenic, and non-integrating. Successful differentiation into clinically-useful MN fates requires good manufacturing practice during the initial steps of iPSC-generation, neuralization, and neural precursor induction.

EBs are clusters of iPSCs or ESCs representing all three germ layers, and an ectodermal (neural) fate is the default specification without extrinsic morphogen treatment. Early MN differentiation methods used EB generation as a crucial first step in neural induction. These methods used a variety of neurotrophic factors to initiate neuralization and to culture neural precursors. Neuralization can be initiated by detaching PSCs from pluripotent media (which contain the factors leukemia inhibitory factor and bFGF), thus causing a termination of pluripotency and generating aggregate embryoid bodies (EB) (Hu and Zhang, 2009). Protocols developed by Hu and Zhang (2009), Karumbayaram et al. (2009), Hester et al. (2011), Amoroso et al. (2013), and Maury et al. (2015) rely on the formation of EBs as the initial step to MN differentiation, with each protocol having unique advantages and disadvantages. Protocols for iPSC-MN generation are summarized in Table 1. In their protocol, Hu and Zhang (2009) generated EB by removing ESCs from mouse embryonic fibroblast feeder culture, mechanically selecting neural colonies, and generating PAX6+ neuroepithelial rosettes in 10 days. After 2 weeks, rosettes expressed SOX1, PAX6, OTX2, and FOXG1 – suggesting a forebrain precursor identity. After removal from ESC medium, cells were cultured in serum-free neural differentiation media in the absence of any morphogens. Thus, neuralization, the initial step in MN differentiation, can occur without any morphogens or neurotrophic factors. Karumbayaram et al. (2009) similarly derived EBs from iPSCs by removal from pluripotent media (containing leukemia inhibiting factor and bFGF) and treatment with collagenase or trypsin/EDTA to break up colonies and increase *in vitro* morphogen coverage. They combined the steps of neuralization and ventralization by culturing iPSC-derived EBs for 1 week before treating them with RA and Purmorphamine (Shh agonist) for one additional week, thus generating spinal precursors expressing PAX6, POU class 3 homeobox 2 (POU3F2), and SOX3 in 2 weeks. Hester et al. (2011) similarly generated neural precursors by forming EBs from iPSCs within 10 days. Amoroso et al. (2013) optimized neuralization by treating iPSCs with ROCK inhibitor Y27632, bFGF, SB435142, and LDN193189. Thus, they combined dual-SMAD inhibition with EB formation, simultaneously inducing a neural, and spinal fate (dual-SMAD inhibition represses mesodermal-lineage-inducing TGFβ-family proteins). After 3 days, EBs were switched to a neural induction medium containing N2 supplement, heparin, penicillin/streptomycin, non-essential amino acids, and L-glutamine. On day 5, spinal precursors were generated by treatment with RA, ascorbic acid, and BDNF. Maury et al. (2015) used a similar approach to forms EBs with dual-SMAD inhibition by treating iPSCs with SB431542 and LDN193189. These protocols demonstrate an early convention for generating neural precursors: that of EB formation through spontaneous formation, supplementation with neural media, or combination with dual-SMAD inhibition.

The formation of EB remains a concern to generating clinically-relevant iPSC-NPCs or iPSC-MNs. Although effective, EBs pose certain challenges, including contamination with non-neural cells, variation between cell lines, scalability, and cost (Van Winkle et al., 2012; Pettinato et al., 2015). Multiple MN protocols have aimed to avoid EBs, with dual-SMAD inhibition performed on adherent culture emerging as a favorite. Chambers et al. (2009) developed a protocol for NPC and MN differentiation from lentiviral-transduced-iPSCs without the use of EBs. After dissociating PSCs into single cells and plating on matrigel, dual-SMAD inhibition was used (Noggin and SB431542), and NPCs were generated in 11 days with a yield of 82%. Neural induction was observed by day 5, with PAX6 expression by day 7 and NES expression by day 11. Neural precursors assumed a forebrain identity by expressing OTX2 and FOXG1. Qu et al. (2014) also developed a highly efficient and chemically defined protocol for iPSC-MN generation on adherent culture. iPSCs were cultured in E8 media on fibronectin, vitronectin, and laminin before adding compound C, which is a dual-SMAD inhibitor of TGFβ-family proteins (Qu et al., 2014). This generated PAX6+ NPCs with an efficient of ∼88% in 5–6 days. Du et al. (2015) used a combination of small molecules and dual-SMAD inhibition to generate NPCs. iPSCs were treated with CHIR-99021 (Wnt agonist), SB431542, and DMH1 – thus combining the steps of neuralization and caudalization to form homogenous neuroepithelial precursors in a single chemically-defined step (Du et al., 2015). Dual-SMAD inhibition is thus a widely used approach to generating NPCs, both using EB and adherent culture methods.

More recent protocols have improved upon previous advances by generating NPCs in a chemically-defined fashion. Lukovic et al. (2017) devised an xeno-free, feeder-free, and EB-free method of differentiating iPSCs into NPCs and ultimately MNs. Using an adherent approach with a simple medium containing insulin, taurine, transferring, and sodium selenite, cells were grown on human foreskin fibroblasts, generating rosette-like neural progenitors in 7 days with >98% efficiency. This group also managed to reduce costs, yielding an overall average price of ∼$100 per 500 ml of neural precursors. However, a primary disadvantage of this method is the mechanical, laborious isolation of neural precursors. Additionally, iPSCs used by this protocol were generated through pMX retrovirus (integrating virus), which is not clinically suitable. Goparaju et al. (2017) used a synthetic mRNA technique with dual-SMAD inhibition and neurotrophic factors to generate MNs. They used the synthetic mRNA transcription NGN1/2/3 and neuronal differentiation 1/2 factors (NEUROD1/2) combined with RA, B27, forskolin, SB431542, and dorsomorphin (Goparaju et al., 2017). Neuralization was measured as early as 24 h after transfection by βIII-tubulin expression, with 85–90% efficient NPC generation on day 5. By avoiding the generation of EB and animal products, these recent methods provides for chemically-controlled, footprint-free NPC generation.

### Differentiation of Motor Neuron Progenitors and General Motor Neurons

After neuralization is induced and NPCs are formed, additional specification takes place to commit cells to a lower MN lineage. Caudalization and ventralization take place to generate a spinal and anterior, ventral MN character, respectively. These MN-committed progenitors most commonly express OLIG2, NGN1/2, NKX6.1, NKX6.2, and PAX6. Once maturation takes place, general MNs express the canonical markers HB9, ISL1/2, and LHX3. The most commonly employed methods to generate MN-committed progenitors and mature MNs include treating NPCs with RA, Shh pathway agonists, and neurotrophic factors.

Early MN differentiation protocols used a wide variety of small molecules, temporal adjustments, and neurotrophic factors to generate cells committed to a MN fate (Table 1). After generating NPCs, Hu and Zhang (2009) initiated treatment of RA on day 10, Shh/B27 on day 15, and neurotrophic factors on day 28 (Hu and Zhang, 2009). MN progenitors were noted with >60% efficiency on day 28, with mature HB9 + MNs appearing on day 35 with >40% efficiency. The authors note the importance of breaking up colonies into smaller components to allow more treatment penetration into dense colonies. This can be achieved manually or with proteases like accutase, which contain proteolytic enzymes. Karumbayaram et al. (2009) first generated NPCs by forming EBs and then treating with RA and Purmorphamine. They formed MN progenitors by continuing RA/Purmorphamine treatment for an additional week in the presence of ciliary neurotrophic factor (CNTF), BDNF, and glial cell line derived neurotrophic factor (GDNF) – ultimately yielding Nkx6.1 and Olig2 expressing MN progenitors in 21 days (Karumbayaram et al., 2009). MNs were generated by day 40 and expressed HB9, ISL1, βIII-tubulin, and ChAT with ∼30% efficiency (Table 1). Similarly, in addition to RA, Purmorphamine, and SAG, Amoroso et al. (2013) added the neurotrophic factors IGF-1, GDNF, and CNTF, B27 on day 17 to generate MNs expressing HB9, Isl1, Isl2 (day 21) with ∼50% efficiency. Of particular interest, Amoroso et al. (2013) hypothesized that different ventralizing agonists, commonly employed in inducing a MN fate, have different differentiation efficiencies. They compared recombinant Shh, human-specific SAG, mouse SAG, and Purmorphamine – finding that a combination of mouse SAG and Purmorphamine to be optimal. Thus, Amoroso et al. demonstrated that the selection and timing of Shh agonist administration is crucial to protocol efficiency. In contrast to the above protocols, Hester et al. (2011) formed EBs and NPCs in 10 days, and then initiated MN progenitor differentiation by treatment of an adenoviral cocktail containing NGN2, ISL1, and LHX3, along with RA, Shh, forskolin, and B27. After 11 days, MNs expressed HB9 and ChAT with 50–70% efficiency. Thus, Hester et al. (2011) succeeded in shortening the duration of differentiation – using an adenoviral cocktail to deliver transcription factors which are characteristic of mature MNs.

Significant advances regarding the optimization of morphogen combinations and temporal treatment have taken place to improve the efficiency and reduce the duration of MN differentiation (Table 1). However, this resulted in significant variability between protocols. After generating NPCs, Chambers et al. (2009) treated cells with BDNF, ascorbic acid, Shh, and RA to generate MNs, which expressed ISL1 and HB9 on day 19. To form MN progenitors, Maury et al. (2015) used the following combination of small molecules: Chir-9902 (Wnt agonist), RA, and FGF2 in the presence of smooth agonist to generate OLIG2+ and NKX6.1 + MN progenitors. To generate mature MNs, DAPT was added, ultimately yielding MNs in 14 days with 77% efficiency. Of particular importance, the authors note the importance of optimizing the timing of small molecule treatment. Qu et al. (2014) also employed strict temporal and media control by adding RA, Shh, and neurotrophic factors on day 3, 6, and 9, respectively. MNs were observed by day 20 with ∼30–52% efficiency. Du et al. (2015) treated NPCs with CHIR, SB431542, DMH1, RA, and Purmorphamine to yield MN progenitors with a yield of >90%. Mature MNs were generated after CHIR, SB431542, and DMH were removed, RA was increased, and Purmorphamine was reduced. After an additional 6 days, 90% of MNs expressed HB9. These protocols generate >90% pure MN progenitors and mature MNs, highlighting the efficient nature resulting from morphogen optimization. As a result, significant diversity exists in the combinations of small molecules and treatment timing that affect the efficiency or duration of MN differentiation protocols.

Recent methods further increased efficiency and clinical-applicability, using synthetic mRNA, xeno-free, feeder-free, and EB-free methods of MN generation. After 7 days of synthetic mRNA induction, Goparaju et al. (2017) noted HB9+, ChAT+, and ISL1+ MNs with 94–100% efficiency. Lukovic et al. (2017) used an insulin containing media to generate NPCs, and on day 21, they treated precursors with RA, bFGF, triiodothyronine hormone 21, and EGF. On day 49, ISL + MNs were generated, thus establishing a clinically applicable protocol for MN generation. These most recent protocols have the highest efficiency and lowest duration, and thus have advantages over earlier methodologies.

The above protocols represent an evolution of understanding and generating iPSC-derived neural cells of the MN-lineage *in vitro*. While early protocols relied on expensive, inefficient, and lengthy methodology, recent advances have allowed for the large-scale generation of functionally mature MNs – using integration-free, feeder-free, chemically-controlled, highly efficient (>98%), and rapid (∼7 days from iPSC to MN) experimental strategies (Goparaju et al., 2017; Lukovic et al., 2017). Future clinical trials could utilize these most recent protocols to generate NPCs and MN-committed cells from iPSCs and transplant these cells into spinal lesions.

### Differentiation of Motor Neuron Subtypes

To date, the majority of iPSC-MN methods have generated generic spinal MNs (usually of a cervical subtype identity), which express canonical markers of MN-lineage – HB9, ISL1, ISL2, and LHX3. However, the differentiation of PSCs into MN subtypes, each expressing unique gene signatures along the spinal rostra-caudal axis, has received significantly less attention. This being said, several studies have succeeded in manipulating MN subtypes during their differentiation.

In their study, Amoroso et al. (2013) generated iPSC-MNs of the LMC subtype. The small molecules RA, SAG, and purmorphamine were used to drive LMC MN generation in a 3-week timeframe, with an efficiency of ∼50%. Of the MNs generated, ∼80% belonged to the medial LMC subtype, with others belonging to the lateral LMC subtype. LMC MNs were confirmed with high FOXP1 expression, a known marker for this subtype. The authors hypothesize that LMC MNs were generated due to HOX gene control of FOXP1, which specifies brachial-lumbar fates (Dasen et al., 2008), or the inhibition of Wnt signaling, which is known to contribute to MMC MNs (Agalliu et al., 2009). Thus, this protocol can be used to generate lateral or medial LMC MNs.

Some MN subtype derivation methods have primarily focused on ESCs rather than iPSC to generate LMC, MMC, and PMC MN subtypes. Patani et al. (2011) studied the effects of RA on neuralization, caudalization, and ventralization to generate MMC MNs, which are responsible for innervating axial musculature. Interestingly, Patani et al. (2011) identified unique RA-dependent and RA-independent pathways, which lead to differing MN subtypes. RA, synthesized by neighboring MN populations in the spinal cord, contributes to a rostral identity, particularly of a LMC MN subtype (Guidato et al., 2003). By inducing MNs independent of RA, primarily MNs of the MMC fate were generated. Thus, MNs may be induced in the absence of RA, which can play a driving role in MN subtype determination. In 2014, Machado et al. (2014) used ESCs to generate MNs with a PMC subtype identity. PMC MNs, which maintain respiration by innervating the diaphragm, are determined by three markers: POU3F1, HOXA5, and Notch. Of particular interest, they found cadherin 10 (CDH10) to be associated with PMC MNs assembling together within groups according to their group identity *in vitro*. Peljto et al. (2010) also succeeded in differentiating ESCs into LMC MN subtypes (Peljto et al., 2010). When these MNs were grafted into embryonic spine, they segregated by subtype and associated closely with their MN subtype *in vivo*. These studies demonstrate that MN subtypes can be patterned from PSCs and associate with their appropriate subtype *in vivo*.

As many of the protocols described thus far have shown, RA is one of the most widely used caudalizing factors in neural induction. Patani et al. (2011) found that RA-dependent and RA-independent differentiation methods lead to different MN subtypes. Further improving on this, Lippmann et al. (2015) used both iPSCs and ESCs to conduct an elegant series of experiments to determine the role of RA in HOX-gene patterning in the differentiation of rostral-caudal MN subtypes. Thus far, it has often been reported that RA induces rostral spinal fates, particularly those associated with caudal hindbrain, and cervical spine (Philippidou and Dasen, 2013). In this study, however, RA was found to induce a transition from neuromesoderm to neuroectoderm and halt HOX rostral-caudal patterning. The authors suggest that previous studies conducted an early termination of HOX gene patterning, resulting in rostral fates. Initially, Wnt, β-catenin (CTNNB1), and FGF were used to induce a stable SOX2+ neuromesodermal progenitor state. At this stage, HOX gene patterning took place, and hindbrain, cervical, thoracic, lumbar, and sacral HOX genes were activated. By varying the timing of Wnt, CTNNB1, and FGF exposure at this stage, progenitors were specified along the rostral-caudal axis. After exposure to RA, progenitors entered the neuroectodermal stage already committed to a positional identity. After maturation, MNs expressed marks characteristic of their earlier patterned fate. Thus, varying the time of exposure to these patterning factors generates different MN populations, including cervical, thoracic, and lumbar MN pools.

Though past and future experimental advances, PSCs have been successfully differentiated into several MN subtypes, namely MMC, LMC, and PMC columns. In addition, recent progress in RA-mediated HOX patterning revealed the role of RA in inducing a neuromesodermal to neuroectodermal transition, thus committing neural precursors to their subtype fate (Lippmann et al., 2015). Future advances in differentiating mature MN subtypes could be promising tools for transplantation in SCI. One of these is the future promise of genetic engineering, which can be used to experimentally refine iterations and stages of MN lineage stem cells until their final subtype fate. Recent advances in genetic engineering, using gene knock-in/out techniques, have revealed previously underappreciated roles of transcription factor regulation in the ventral spinal cord, especially that of Notch1 regulation by Nkx6.1 (Li et al., 2016). Genetic modification, in the context of engineered drug resistance, has also been used to enrich for specific MN subpopulations and may be used in future studies to precisely select for desired subtypes (McCreedy et al., 2012). Thus, the promise of genetic engineering may be used to further explore and generate MN subtypes, especially in the context of novel treatments for SCI.

### Optimizing Research Design and Choosing the Correct MN Differentiation Protocol

Multiple factors should be considered when selecting an iPSC-MN differentiation protocol. The protocols reviewed here each have advantages and disadvantages based on the goals of a proposed study. In particular, research groups should weigh factors that include but not limited to clinical relevance, viral integration, duration of iPSC-MN differentiation, cost-effectiveness, efficiency, scalability, MN subtype generated, and if MNs have previously been confirmed with electrophysiology or *in vivo* grafting.

First of all, for clinical applications, the recent protocol developed by Lukovic et al. (2017) provides an xeno-free, feeder-free, and EB-free method of MN differentiation from iPSCs. In addition, this protocol minimizes costs, with neural precursors costing ∼$100 per 500 ml. The group used an adherent approach along with insulin-based media to grow NPCs on foreskin fibroblasts, later treating NPCs with RA, bFGF, triiodothyronine hormone 21, and EGF. Overall, MNs were generated in 49 days (iPSC to MN), making this one of the lengthier MN differentiation protocols. MNs were confirmed with electrophysiology as well as *in vivo* grafting (mouse striatum). However, iPSCs were generated by using a pMX integrating viral transfection. As a result, combining this protocol with iPSCs generated by non-integrating means would further increase clinical applicability. For research groups interested in clinical applications with a shorter protocol duration, the method developed by Goparaju et al. (2017) generates mature MNs in 7–14 days with 94–100% efficiency through chemically-defined synthetic mRNA transfection. This protocol is robust and integration-free, and mature MNs were confirmed with electrophysiology and calcium imaging. Thus, these two most recent protocols provide clinically-relevant protocols for iPSC-MN generation.

Secondly, for applications prioritizing scalability, protocols developed by Qu et al. (2014), Du et al. (2015), and Maury et al. (2015) provide for efficient, high-throughput iPSC-MN differentiation. Both Qu et al. (2014) and Du et al. (2015) used an adherent dual-SMAD inhibitory approach to generate mature MNs in 20 days with ∼30–52% efficiency and 28 days with ∼90% efficiency, respectively. In contrast, Maury et al. (2015) used an EB based method in combination with dual-SMAD inhibition to generate mature MNs in 14 days with 77% efficiency. This last protocol holds potential for high-throughput, automatable applications due to its 384 well-plate design. Research groups interested in automation and high-throughput MN differentiation should consider these methodologies.

Finally, for applications prioritizing specific MN subtypes, research groups may consider the Amoroso et al. (2013) protocol to specifically generate lateral or medial LMC MNs. For other MN subtypes, the protocol designed by Lippmann et al. (2015) may be considered. This protocol in particular highlights the previously unknown role of RA in cementing rostral-caudal identity. Research groups may generate diverse MN subtypes simply by varying the timing of Wnt, β-CTNNB1, and FGF exposure. When the desired subtype identity is imprinted, cells may be treated with RA to relatively permanently induce neuroectodermal fate of a specific rostral-caudal subtype. Thus, multiple subtypes of MNs may be generated from iPSCs for use in downstream applications, such as transplantation in SCI.

## Applications in Spinal Cord Injury

### Neural Precursor Transplantation

Direct transplantation of undifferentiated iPSCs into SCI lesions is not tenable because of the significant risk of teratoma formation, as well as a lack of direction regarding cellular fate. As a result, over the past decade, iPSC-derived cells already committed to a neural fate (but in various stages of neural lineage) have been transplanted in models of SCI (Tsuji et al., 2010; Nori et al., 2011; Kobayashi et al., 2012; Ruzicka et al., 2017). Neural precursors are used for their *in vivo* differentiation capacity into MNs and glia, thus replacing damaged axonal tracts and exerting a variety of neurotrophic, remyelinating, and relay-forming effects (Nori et al., 2017). For these reasons, the transplantation of neural precursors and other stem cells may be beneficial in MN diseases where the best possible site of transplantation may not be apparent (e.g., ALS, severe SCI) precisely because of these paracrine, neurotrophic secretory effects. In this section, we explore the application of transplanting stem cell derived NPCs, MN progenitors, and mature MNs in SCI.

While iPSC-NPCs have beneficial effects, the hostile microenvironment of SCI remains a challenge to MN growth. Immune and ischemic processes combine forces to recruit cytokines/chemokines, generate free-radicals, and stunt nascent cell development. Neither acute nor chronic stem cell transplants have yet been largely successful due mainly to the early inflammatory response, which is particularly damaging to cell grafts, and formation of the glial scar, which inhibits axonal regrowth. Thus, transplants occurring during the subacute phase, considered the optimal timeframe, have been shown to be more effective (Nishimura et al., 2013; Cheng et al., 2017; Lane et al., 2017; Nori et al., 2017). In the subacute phase, M2 macrophages, which have immune-mediating characteristics, are recruited to mitigate inflammation. Additionally, the glial scarring has not yet fully formed. Functional recovery after transplantation has been noted in many different stages of SCI; however, transplantation in the subacute phase yields optimal results (Nori et al., 2017). Thus, the precise timing of iPSC-NPC transplantation following SCI remains an important clinical consideration for individuals with SCI.

iPSC-NPC transplants in SCI have largely yielded encouraging results. When compared with other sources, including MSCs and fetal spine cells, iPSC-derived neural progenitors show greater functional recovery after SCI (Ruzicka et al., 2017). Nori et al. (2011) reported that iPSC-derived neurosphere transplantation, consisting of neural stem cells and progenitors, yielded differentiation into neurons, astrocytes, and oligodendrocytes—synapsing with host neurons, migrating into the spinal cord, and resulting in significant locomotor functional recovery. Nearly half of the graft cells (49.1%) differentiated into upper MNs, which were predominantly GABAergic. And astrocytes, which expressed vascular endothelial growth factor (VEGF), were concluded to have promoted angiogenesis and tissue sparing. Fewer cells differentiated into lower MNs. Kobayashi et al. (2012) also reported that iPSC-NPCs survived and differentiated in a marmoset model of SCI, promoting locomotor recovery. Other studies have also yielded similar positive results (Tsuji et al., 2010; Lu et al., 2014a; Romanyuk et al., 2015; Salewski et al., 2015), while others have confirmed successful NPC integration into both healthy and injured spinal cord (Lepore and Fischer, 2005; Lu et al., 2014b; Sareen et al., 2014). Recent clinical trials using ESC-derived NPCs have yielded positive results for a small number of individuals, but iPSC-NPCs have yet to be tested in clinical trials (Curtis et al., 2018; Nagoshi and Okano, 2018). In addition, a recent meta-analysis of iPSC-derived NPC transplants, surveying six randomized controlled preclinical trials, showed significant locomotor recovery, especially with compression injuries, moderate cell counts (5 × 10^5^), intraspinal routes of administration, and neural precursor transplants (Qin et al., 2018). Another recent meta-analysis of 12 iPSC-NPC studies also showed significantly improved locomotor recovery (Yousefifard et al., 2016). Taken together, these results highlight the significant potential of neural precursors to integrate into the spinal cord and promote locomotor recovery.

Studies have shown that one of the primary benefits of iPSC-NPC transplantation is integration with, and nourishment/replacement of, upper and lower MNs. After iPSC-NPC transplantation in rat SCI, Romanyuk et al. (2015) observed the formation of nascent HB9+cholinergic lower MNs in the ventral spinal cord. The MNs communicated with astrocytes and associated with their correct tissue-specific phenotype, ultimately helping salvage white and gray matter, and increasing locomotor function. Similarly, other studies have reported the restoration of upper MNs after iPSC-NPC transplantation. Fujimoto et al. (2012) reported that iPSC-derived NPCs, in the form of neuroepithelial-like stem cells, worked in concert with surviving endogenous neurons to reconstruct the corticospinal tract in a mouse SCI model. Recently, Mark Tuszynski’s (2016) group reported that both ESC-derived and iPSC-derived NPCs restored corticospinal tract structure when grafts are of a caudalized, spinal fate (Kadoya et al., 2016). Existing damaged corticospinal tracts were able to connect to grafts regardless of graft maturity or stage of development, resulting in improved gripping ability. Similarly, other groups have found neural precursors to differentiate *in vivo* and graft into their correct domains, forming appropriate synapses according to their identity (Dulin et al., 2018). For these reasons, iPSC-derived NPCs are a promising therapeutic strategy for *in vivo* MN differentiation, integration, and replacement following SCI.

### Motor Neuron Progenitor Transplantation

To date, a majority of studies have investigated the therapeutic role of NPCs in SCI. Some studies, however, have described transplantation of cells already committed to the MN lineage by transplanting neuronal progenitor cells. In theory, transplanting MN-committed progenitors could more effectively target and replace specific damaged MN tracts/columns, possibly even reducing the risk of teratoma formation after transplantation. This is because transplanting cells already committed to a MN lineage more closely resembles the damaged MNs in SCI. These studies have observed some positive effects in SCI models, such as increased functional recovery or greater sparing of endogenous neurons (Rossi et al., 2010; Wyatt et al., 2011), but questions remain over whether this is due to neuronal replacement itself or neurotrophic signals released by the MN progenitors. No cell migration cranially or caudally was observed in either of these studies, suggesting that cells at a more differentiated state may have less capability to migrate (vs. NPCs, which have been found to migrate as noted previously). In addition, one of these studies (Rossi et al., 2010) found that implanted neuronal stem cells result in a 15-fold decrease in the expression of inflammatory and pro-apoptotic markers, suggesting that motor neuronal stem cells may have immunomodulatory effects.

In some cases, research groups have found conflicting results. Interestingly, some groups have found difficulty differentiating rat-derived neuron-restricted precursors into mature MNs in the harsh SCI microenvironment in rats (Cao et al., 2002; Tarasenko et al., 2007; Rossi et al., 2010; Kadoya et al., 2016). Some of these studies report that *in vitro*, MN or neuronal progenitors differentiate into neurons, but *in vivo*, they predominantly differentiate into glia. This could be due to the relatively lower plasticity of MN progenitors than NPCs, which differentiate into mature MNs in SCI (Fujimoto et al., 2012; Lu et al., 2014b; Romanyuk et al., 2015). Mark Tuszynski’s group resolved this by transplanting embryonic mouse-derived neuron-restricted precursors into a mouse SCI model, ultimately also showing that functional recovery occurred irrespective of graft maturity. They also showed that cells restricted to a neuron lineage were required for corticospinal regeneration, rather than glial precursors alone. Along these same lines, other groups report that transplanted cells often differentiate into a diverse mix of interneurons and ventral lower MNs that express classical markers of both early and late phase neurogenesis (Kumamaru et al., 2019), thus making precise *in vivo* cell replacement strategies challenging. Future studies will need to elucidate the microenvironmental mechanisms behind why NPCs and MN progenitors can be driven toward relatively pure MN fates *in vitro*, but become a mix of glial or interneuron fates when transplanted *in vivo*. Regardless of these challenges and the specific mechanism yet to be elucidated, MN-progenitor transplants in SCI models have also been largely restorative (Rossi et al., 2010; Wyatt et al., 2011; Kadoya et al., 2016).

### Targeting Motor Neuron Subtypes in Spinal Cord Injury

While iPSC-derived post-mitotic MNs are able to integrate effectively into the healthy spinal cord (Amoroso et al., 2013; Su et al., 2013; Davis-Dusenbery et al., 2014), the harsh “immune-ischemic microenvironment” of SCI makes transplantation of already mature cells challenging. This is due to myelin associated proteins inhibiting axonal regrowth, glial scarring, and the significantly lower plasticity of post-mitotic neurons (Busch and Silver, 2007; Barnabe-Heider and Frisen, 2008; Ruff et al., 2012). For these reasons, the transplantation of specific iPSC-derived MN subtypes into SCI lesions is currently untenable. The significant progress made in deriving individual MN subtypes from iPSCs remains subject to discovering novel methods of supporting post-mitotic MN survival in the unfavorable microenvironment of SCI (Davis-Dusenbery et al., 2014; Iyer et al., 2017; Lane et al., 2017). Because MN subtypes have differential vulnerabilities in SCI, targeting individual MN subpopulations is critical to developing effective, personalized treatments for SCI.

This being said, we have identified several ways that iPSC-derived cells or exogenous treatments may be used to target individual MN subtypes in SCI. First, previous studies have reported that mature MNs may be able to survive in SCI lesions if co-transplanted with other cells like olfactory ensheathing cells, which release neurotrophic factors and promote axonal growth (Salehi et al., 2009; Ekberg and St John, 2015). For example, the PMC MN subtype, innervating the diaphragm, is an especially relevant MN pool in SCI modeling (Kastner and Gauthier, 2008). Recent studies have used NPC populations enriched in V2a interneurons to target PMC MN pools, showing significantly more respiratory improvement than NPCs alone (Zholudeva et al., 2018). Other groups have used iPSC-derived astrocytes to target lesioned PMC MNs, resulting in reduced denervation at the diaphragmatic neuromuscular junction (Li et al., 2015). Thus, synergistic effects through co-transplantation of iPSC-derived astrocytes or NPCs in combination with olfactory ensheathing cells or interneurons may aid the survival of MN subtypes. Second, *in vivo* programming strategies taken from the current understanding of iPSC-MN generation may guide differentiation toward desired MN subtypes (Peljto et al., 2010). Endogenous factors, including RA and Wnt signaling, may be manipulated to generate different MN subtypes along the rostral-caudal axis (Lippmann et al., 2015). Similarly, resident spinal cells, including astrocytes, may be reprogrammed to MNs *in vivo* (Su et al., 2014). Thus, MN subtypes could be generated *in vivo* or *in situ* to replace lost neuronal tracts/columns. Third, recent advances in the field of biopolymer scaffolding or microenvironment control may be utilized to increase grafting efficiency of MN subtypes (Abati et al., 2018). Fourth, resident spinal MN subtypes could be targeted by exogenous treatments. Anderson et al. (2018) recently targeted descending propriospinal neurons using temporally and spatially controlled administration of neurotrophic factors in SCI rat models. In addition, the authors found that neuron subtypes have unique requirements for activation and regeneration, with different subtypes requiring different treatment strategies (Duan et al., 2015). As a result, it may be possible that some MN subtypes are better targeted with iPSC-derived cell replacement while others should be targeted with other strategies. Thus, MN subtypes should continue to be targeted following SCI by co-transplantation with iPSC-derived cells, *in vivo* directed differentiation, optimizing scaffolding and microenvironmental manipulation, and treatment with neurotrophic factors.

## Conclusion and Future Directions

The transplantation of iPSC-derived MNs in SCI remains in its infancy. While promising advances have taken place, especially with regard to the significant body of evidence showing functional recovery in SCI models after NPC transplantation, clinical translation remains challenging. Variability in stem cell sources, experimental protocols, and ambiguity regarding the most clinically effective and safe procedures remain significant hurdles to enrolling and treating individuals with SCI in future clinical trials. Further, the heterogeneous nature of SCI itself poses a challenge for one-size-fits-all therapies. Thus, significant improvements in protocol standardization as well as our understanding of SCI pathophysiology are needed.

Future advances in mechanistically understanding CNS development, stem cell induction, and SCI pathophysiology are critical. Targeting specific MN subtypes, especially those most vulnerable following SCI, is a previously underappreciated therapeutic strategy. Future stratification of individuals and injury types according to the individual MN subpopulations affected will allow for personalized stem cell transplantation. In addition, advances in differentiating additional MN subtypes, particularly the differentiation of upper MNs, are needed to effectively target upper MN tracts. This is because upper MN tracts (corticospinal, reticulospinal, tectospinal, rubrospinal, and vestibulospinal) are differentially vulnerable during SCI. Significant advances need to be made regarding *in vitro* differentiation protocols before upper MN precursors may be transplanted into MN columns inn spinal lesions. In addition, researchers have yet to fully take advantage of advances in RA-mediated HOX patterning to generate neural precursors with distinct identities along the rostral-caudal axis for transplantation following SCI. These advances would allow for neural precursors to be transplanted with a MN subtype fate, leading to specific targeting of particular spinal lesions (depending on both their location along the rostral-caudal axis and which MN subtypes are affected). As these new methods evolve, the use of integration-free, feeder-free, and virus-free reagents is crucial at every step for streamlined clinical applications.

iPSC-derived neural-committed cells are powerful therapeutic tools in stem cell transplantation for SCI. While previous stem cell sources (ESCs and MSCs) continue to be dogged by ethical, immunogenic, and clinical concerns, iPSCs are an alternative with significant advantages. iPSCs may be differentiated into MNs and different MN subtypes by a wide variety of methods. These protocols have undergone significant revision and improvement, and the most recent methods strive to be as translatable as possible. Significant technological and methodological advances have taken place that may now allow for easier clinical translation. Further, the recent differentiation strategies that generate individual MN subtypes should continue to become more relevant as personalized stem cell transplantation becomes reality. Future advances are needed to take advantage of these methods and develop novel treatment strategies able to target individual MN subtypes – performed with the ultimate goal of restoring motor function and quality of life for individuals with SCI.

## Author Contributions

MT developed the idea in detail, reviewed the literature, and wrote the manuscript. GL designed the original project idea and guided the development of the review throughout the writing process. BD provided valuable feedback and helped to revise the manuscript. RF provided the leadership for the project and furnished the valuable feedback for revising the manuscript.

## Conflict of Interest Statement

The authors declare that the research was conducted in the absence of any commercial or financial relationships that could be construed as a potential conflict of interest.

## Abbreviations

ALS: amyotrophic lateral sclerosis
BDNF: brain derived neurotrophic factor
bHLH: basic helix-loop-helix
BMP: bone morphogenetic protein
ChAT: choline acetyltransferase
c-Myc: MYC proto-oncogene
CNTF: ciliary nerutrophic factor
CTNNB1: β-catenin 1
Cre-loxP: cyclization recombinase locus of X over P1
DMH1: dorsomorphin homolog 1
EB: embryoid bodies
EGF: epidermal growth factor
ESC: embryonic stem cell
FGF: fibroblast growth factor
FOXG1: forkhead box G1
FOXP1: forkhead box P2
GDNF: glial line derived neurotrophic factor
HB9: motor neuron and pancreas homeobox 1
HD: homeodomain
HMC: hypaxial motor column
ISL1: ISL LIM homeobox 1
ISL2: ISL LIM homeobox 2
iPSC: induced pluripotent stem cell
KLF4: kruppel like factor 4
LHX3: LIM homeobox 3
LMC: lateral motor column
MMC: medial motor column
MN: motor neuron
MSC: mesenchymal stem cell or multipotent stem cell
NES: nestin
NGN1: neurogenin 1
NGN2: neurogenin 2
NKX2.2: NK2 homeobox 2
NKX6.1: NKX homeobox 1
NPC: neural precursor cells
OCT4: octamer-binding transcription factor 4
OLIG2: oligodendrocyte transcription factor 2
OTX2: orthodenticle homeobox 2
PGC: preganglionic column
PMC: phrenic motor column
PSC: pluripotent stem cell
POU3F1: POU class 3 homeobox1
RA: retinoic acid
SAC: spinal accessory column
SAG: smoothened agonist
SCI: spinal cord injury
Shh: sonic hedgehog
SMAD: mothers against decapentaplegic-family proteins
SOX1: sex determining region Y box 1
SOX2: sex determining region Y box 2
TGF β: transforming growth factor beta
Wnt: wingless/integrated.
